# The PD-1/B7-H1 Pathway Modulates the Natural Killer Cells versus Mouse Glioma Stem Cells

**DOI:** 10.1371/journal.pone.0134715

**Published:** 2015-08-12

**Authors:** Bo Yuan Huang, Yi Ping Zhan, Wen Jing Zong, Chun Jiang Yu, Jun Fa Li, Yan Ming Qu, Song Han

**Affiliations:** 1 Department of Neurosurgery, Beijing San Bo Brain Hospital, Capital Medical University, Beijing, China; 2 College of traditional Chinese Medicine, Capital Medical University, Beijing, China; 3 Department of Neurosurgery, Brain Sciences Institute of Beijing, Capital Medical University, Beijing, China; Swedish Neuroscience Institute, UNITED STATES

## Abstract

**Purpose:**

Glioblastoma multiforme (GBM) is the most malignant primary type of brain tumor in adults. There has been increased focus on the immunotherapies to treat GBM patients, the therapeutic value of natural killer (NK) cells is still unknown. Programmed death-1 (PD-1) is a major immunological checkpoint that can negatively regulate the T-cell-mediated immune response. We tested the combination of the inhibiting the PD-1/B7H1 pathway with a NK-cell mediated immune response in an orthotopic mouse model of GBM.

**Methods and Materials:**

Mouse glioma stem cells (GL261GSCs) and mouse NK cells were isolated and identified. A lactate dehydrogenase (LDH) assay was perfomed to detect the cytotoxicity of NK cells against GL261GSCs. GL261GSCs were intracranially implanted into mice, and the mice were stratified into 3 treatment groups: 1) control, 2) NK cells treatment, and 3) PD-1 inhibited NK cells treatment group. Overall survival was quantified, and animal magnetic resonance imaging (MRI) was performed to determine tumor growth. The brains were harvested after the mice were euthanized, and immunohistochemistry against CD45 and PCNA was performed.

**Results:**

The mouse NK cells were identified as 90% CD3^-^ NK1.1^+^CD335^+^ by flow cytometric analysis. In the LDH assay, the ratios of the damaged GL261GSCs, with the E:T ratios of 2.5:1, 5:1, and 10:1, were as follows: 1) non-inhibited group: 7.42%, 11.31%, and 15.1%, 2) B7H1 inhibited group: 14.75%, 18.25% and 29.1%, 3) PD-1 inhibited group: 15.53%, 19.21% and 29.93%, 4) double inhibited group: 33.24%, 42.86% and 54.91%. In the in vivo experiments, the mice in the PD-1 inhibited NK cells treatment group and IL-2-stimulated-NK cells treatment group displayed a slowest tumor growth (F = 308.5, P<0.01) and a slower tumor growth compared with control group (F = 118.9, P<0.01), respectively. The median survival of the mice in the three groups were as follows: 1) conrol group: 29 days, 2) NK cells treatment group: 35 days (P = 0.0012), 3) PD-1 inhibited NK cells treatment group: 44 days (P = 0.0024). Immunologic data of PCNA-positive cell ratios and CD45-positive cell ratios of the tumor specimens in the three groups were as follows: 1) control group: 65.72% (PCNA) and 0.92% (CD45), 2) NK treatment group: 27.66% (PCNA) and 13.46% (CD45), and 3) PD-1 inhibited NK cells treatment group: 13.66% (PCNA) and 23.66% (CD45) (P<0.001).

**Conclusion:**

The results demonstrated that blockade of PD-1/B7H1 pathway could promote mouse NK cells to kill the GL261GSCs, and the PD-1-inhibited NK cells could be a feasible immune therapeutic approach against GBM.

## Introduction

Glioblastoma multiforme (GBM) is the most common and aggressive type of primary malignant tumor of the central nervous system [1]. Despite multiple therapeutic approaches, including surgery, radiotherapy and chemotherapy, the prognosis for patients remains dismal, with a median survival of 14.6 months [2]. Traditionally, the main challenges for successfully curing GBM are overcoming the ability of tumor cells to invade the adjacent brain parenchyma as well as the molecular and cellular heterogeneity that underlie their inherent resistance to radiotherapy and chemotherapy. Indeed, there is a growing interest in establishing an effective immunotherapy for GBM by the stimulated immune cells expanded in vitro. Multifarious studies have researched potential candidates for effective immunotherapy of immune cells, such as cytotoxic T lymphocytes (CTLs), dendritic cells (DC), and natural killer (NK) cells [3–5]. A growing number of early clinical trials focused on the CTL-mediated immune response to treat malignancies, such as melanoma [6]. However, these clinical trials required a common precondition, the activation of the T cells, with a prerequisite of the presentation of an antigen to the T-cell receptor (TCR) via the antigen-presenting cell (APC) with a major histocompatibility complex (MHC) molecule.

Among the cytotoxic immune cells, NK cells are the first line of defense in the innate immune system and are supposed to be the most efficient effectors against tumors and pathogens [7]. Tumors and virus-infected cells can usually evade the recognition of CTLs by down-regulating the expression of class I MHC (MHC-I) molecules. However, NK cells, which are stimulated by either altered or lost MHC-I molecules [8], can overcome this “immunologic Achilles' heel”. Moreover, unlike CTLs, NK cells can be directly stimulated without of the requirement of an indispensable antigen presentation via APC. Thus, NK cells are potential candidates as an adaptive immune treatment against malignancies. Moreover, the activation of NK cells is directly and solely regulated and balanced by the stimulatory signals and inhibitory signals [8,9]. It was only after the identification of the co-inhibitory molecules, such as the programmed death-1 (PD-1), that co-inhibitory molecules have come the forefront of the immunological research [10].

PD-1, also called CD279, is a major immunological checkpoint that belongs to the CD28 family. It has an extracellular IgV domain and intracellular tail containing two motifs: an immunoreceptor tyrosine-based inhibitory motif (ITIM) and an immunoreceptor tyrosine-based switch motif (ITSM) [11,12]. The ITIM is thought to mediate inhibitory signals, while the ITSM is responsible for signaling after PD-1 ligation [13]. PD-1 is expressed on various immune cells, such as activated T cells, thymocytes, NK cells and myeloid cells [13–16]. The PD-L1 and PD-L2 ligands are expressed by various tumor cells, including breast cancer, thyroid carcinomas, and GBM [17–19]. Clinical trials have suggested that the expression of PD-L1 (B7H1, also called CD274) by tumors may be an important biomarker of anti-PD-1 efficiency [20]. PD-L1 expressed by tumor cells can engage the PD-1 receptor and induce T-cell exhaustion, and eventually inhibit T-cell activation and proliferation [21]. Thus, the interaction between PD-1 and its ligands, especially PD-L1, may contribute to the immune suppression of the tumor microenvironment, and therefore, blockade of this pathway is a potential approach to promising immunotherapy for cancers [22].

Several previous studies have demomstrated that activated NK cells have significant therapeutic effects against GBM in vivo [23]. However, results from the injection of NK cells into the subcutaneous and intracranial GBM xenograft animal models [23–24] is difficult to apply to the clinical practice. In this study, we investigated whether the blockade of the PD-1/B7H1 immune checkpoint can enhance the activated mouse NK cell-mediated killing of mouse glioma stem-like cells (GL261GSCs) isolated from the GL261 cell line in in vitro experiments. Moreover, with a GL*261GSCs homograft intracranial animal model, we evaluated the anti-tumor effects of PD-1-inhibited mouse NK cells using intravenous injection. The allogeneic immune treatment of PD-1 inhibited mouse NK cells demonstrated a significant therapeutic effect compared to normally activated NK cells.

## Materials and Methods

### Isolation of the GL261GSCs and mouse NK cells

The protocol was approved by the Committee on the Ethics of Capital Medical University Animal Experiments and Experimental Animals Management Committee (permit number: SCXK(京)2012-0001). The YAC-1 and the GL261 cell lines (ATCC) were cultured in RPMI1640 (Life Technologies, USA) containing 10% fetal bovine serum (FBS, Gibco, USA). The GL261GSCs were obtained by the clonal formation method as previously described [25]. Briefly, single-cell suspensions were adjusted to 10^4^ cells/ml and serially diluted to one cell/200 μl. The cells were transferred into a 96-well plate to form clonal spheres. The GL261GSCs were cultured as spheres in serum-free DMEM/F12 medium supplemented with 2% B-27 (Life Technologies, USA), 20 ng/ml epidermal growth factor (EGF, R&D Inc), and 10 ng/ml basic fibroblast growth factor (bFGF, R&D Inc).

Resting mouse NK cells (CD49b^+^, CD3^-^) were gained from C57BL/6 mouse spleens as previously described [26]. Briefly, mouse spleens were aseptically separated, cut into pieces, ground in a cell sieve with phosphate buffered saline (PBS), and washed at intervals. Then, a single-cell suspension was generated. The cells were incubated in a red cell lysis buffer to remove the red cells. After the depletion of CD3^+^ T cells by a CD3 Microbead Kit (Miltenyi Biotec, Germany), the NK cells were positively selected with a CD49b (DX5) Microbead Kit ((Miltenyi Biotec, Germany) according to the manufacturer's protocol. Then the celld were stimulated for 4–8 days in the presence of mouse IL-2 (1000 IU/ml) in RPMI1640 supplemented with 10% inactivated FBS.

### Flow Cytometry

Cells were stained with the following mAbs for phenotypic analysis: FITC anti-mouse CD3 (clone 17A2, Biolegend), APC anti-mouse NKp46 (CD335, clone 29A1.4, Biolegend), and PE anti-mouse NK1.1 (clone PK136, Biolegend). The isotype control IgG was obtained from the Biolegend. Flow cytometry analysis was performed on a BD Biosciences FACSCanto, and the data were analyzed using FlowJo software.

### Immunofluorescence

GL261GSCs spheres were dissociated using Accutise (EMD Millipore, USA) and then seeded into the a laser scanning confocal Petri dish with the RPMI1640 containing 10% FBS. After 6 hours, the 1640RPMI was removed and the cells were washed with the PBS for three times. Then the cells were fixed with 4% paraformaldehyde and blocked with goat serum. Three primary antibodies, CD133 (Miltenyi Biotec, Germany), nestin (R&D system, USA), and B7H1 (CD274, Biolegend, USA) were incubated for 24 h at 4°C followed by detection with the corresponding fluorescent secondary antibodies. The nuclei were counterstained with Hoechst34589 (Santa Cruz Biotechnology, USA). The samples were observed with a confocal laser scanning microscope.

### Cytotoxicity assays

Single-cell suspensions of GL261GSCs and IL-2 stimulated NK cells were prepared at a density of 2.5×10^5^ cells/ml. The Purified anti-mouse CD279 (clone 29F.1A12, Biolegend) and Purified anti-mouse CD274 (clone 10F.9G2, Biolegend) were added into the cell cultures for 24 hours to block the PD-1 on NK cells and B7H1 on GL261GSCs, respectively. Then the cells were plated at specified effector-to-target (E:T) ratios (2.5:1, 5:1, 10:1) into 96-well plates as the following groups: NK cells and GSCs (non-inhibited group), NK cells and anti-B7H1-GSCs (B7H1 inhibited group), anti-PD-1-NK cells and GSCs (PD-1 inhibited group), and anti-PD-1-NK cells and anti-B7H1-GSCs (double inhibited group). The cells were co-cultured for 6 hours in a 37°C incubator. Then, GL261GSCs lysis was detected by the release of lactate dehydrogenase (LDH) using an LDH cytotoxicity assay kit according to the manufacturer's instructions (Promega, USA). The YAC-1 cells, which are the specific targets of mouse NK cells cytotoxicity, were used as a positive control. After a 6 hours incubation, the absorbance was measured at 490 nm with a Multiscan MS microplate reader. Specific lysis of GL261GSCs was calculated as described previously [27]: specific lysis ratio = (experimental release-spontaneous release)/(maximum release-spontaneous release)×100%.

### GL261GSCs mouse model

Seven-week-old wild type (WT) C57BL/6 female mice were maintained in a specific pathogen-free facility. GL261GSCs (5×10^5^ in 5 μl PBS) were intracranially injected into the right hemisphere of mice, using a mouse stereotaxic apparatus at coordinates 1 mm anterior from the bregma, 1 mm lateral, and 3 mm ventral to the surface of the brain and delivered at a rate of 0.2 μl/min over 5 min [28]. Tumor growth was determined with an animal magnetic resonance imaging (MRI) system (PharmaScan 7T, Bruker, Germany) on days 7, 14, 21 and 28. When the first MRI scanning confirmed the tumor formation on the 7th day after the tumor cells implantation, the PD-1 inhibited and non-inhibihted IL-2-stimulated NK cells (5×10^6^, in 100μl PBS) and PBS (negative control) were intravenously injected into the mice containing GL261GSCs tumors once a week for four weeks [29]. The tumor volume was calculated by measuring the MRI of the largest tumor portion and applying the following formula: (width)^2^ x length x 0.5. The mice were euthanized by subcutaneously injected excessive 5% chloral hydrate when they displayed obvious symptoms, such as weight loss > 20% body mass, limbs paralysis or movement disorder, lethargy, and a hunched posture.

### Histology and Immunohistochemistry

Following euthanasia of the mice, brains were harvested and processed for paraffin embedding. Immunohistochemistry against CD45 (Abcam, USA) and PCNA (Abcam, USA) was performed as described previously [30]. The quantification was performed by counting the number of stained cells in ten random fields for each animal.

## Statistical Analyses

The comparisons of GL261GSCs lysis percentage between the groups were performed using one-way ANOVA comparison tests with a Bonferroni correction. The tumor volumes between groups were compared with one-way ANOVA comparison tests. Mouse survival was analyzed by Kaplan-Meier survival curves, and the comparisons of the curves were performed with the log-rank Mantel-Cox test using GraphPad Prism. P values<0.05 were considered statistically significant.

## Results

### Isolation and identification of GL261GSCs stem-like cells

**Fig 1:**
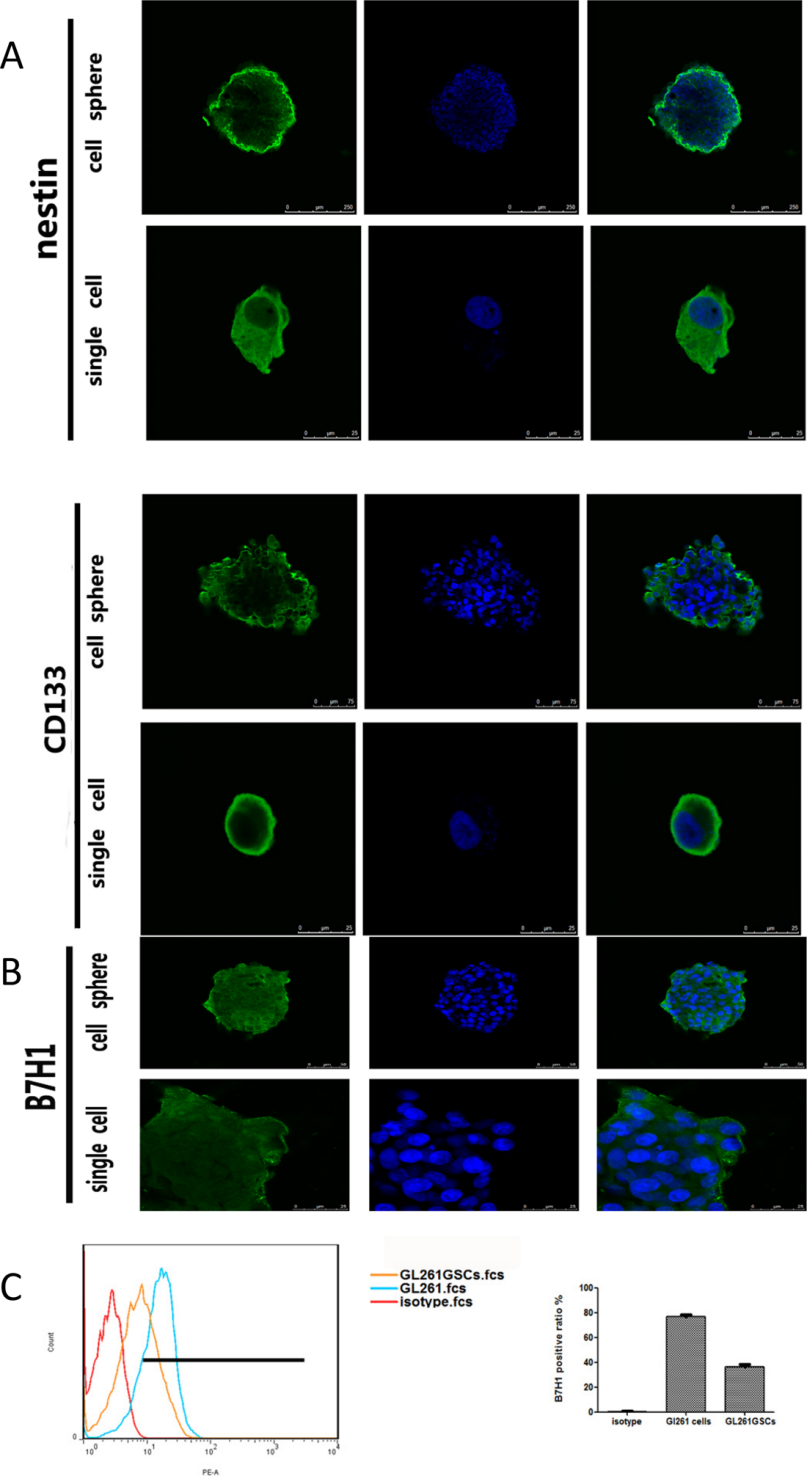
Expression of CD133, nestin and B7H1 on GL261GSCs. (A) GL261GSCs were stained with CD133 and nestin, nuclei were stained with DAPI, positively stained single cell and cell sphere were shown. (B) GL261GSCs stained with B7H1(GFP) were detected by Immunofluorescence. (C) GL261 cells and GL261GSCs were stained with PE-anti-CD274 and detected by flow cytometry.

Using a stringent dilution cloning formation method, GL261GSCs were isolated from the mouse glioma cell line GL261. To identify the immunophenotype of the GL261GSCs, immunofluorescence was performed to analyze the expression of CD133 and nestin. Notably, the GL261GSCs showed statistically significant expression of CD133 and nestin (Fig 1A). To confirm that the PD-1/B7H1 pathway exists in the interaction between the GL261GSCs and mouse activated NK cells, the expression of B7H1 (PD-L1, CD274) was also detected by flow cytometric analysis and immunofluorescence. Compared with GL261 cells, GL261GSCs showed a statistically lower level of B7H1 expression (Fig 1C).

### Isolation of muose NK cells and cytotoxicity against GL261GSCs

**Fig 2:**
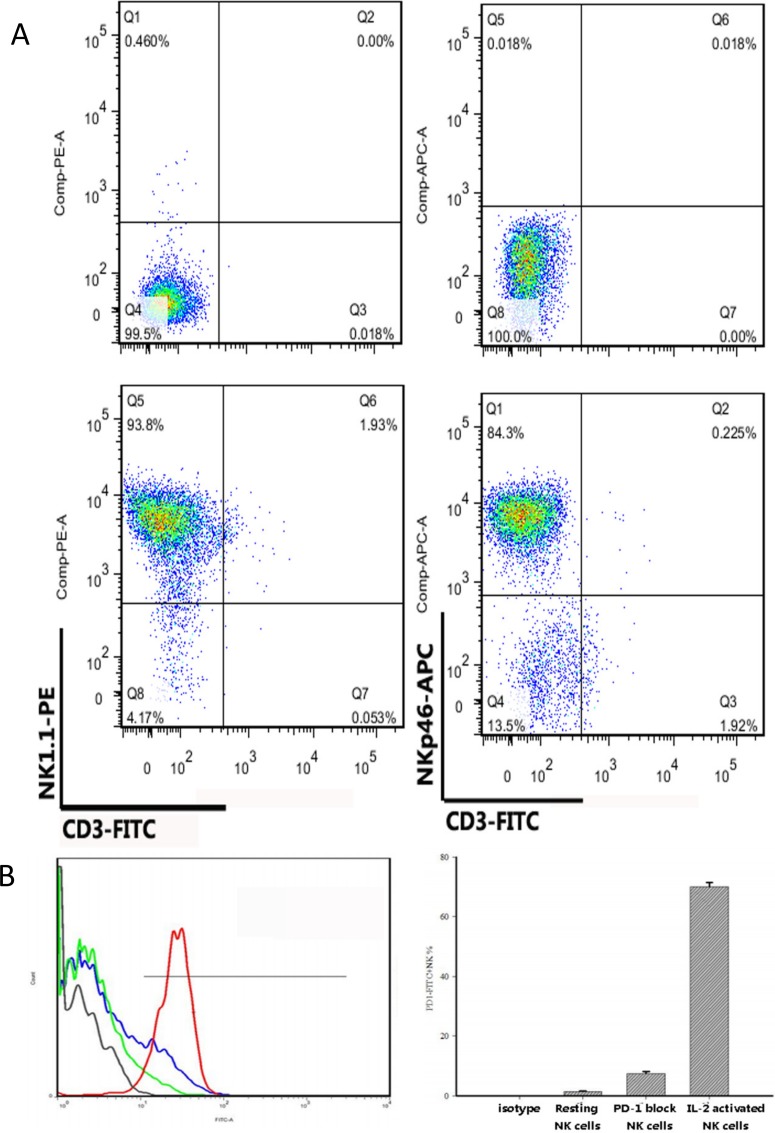
Analysis of mouse NK cells phenotype by flow cytometry. (A) Cells were stained with CD3-FITC, NK1.1-PE and NKp46-APC. The NK cells isolated from mouse spleens were 90% CD3^-^ NK1.1^+^ NKp46^+^. (B) Expression of PD-1(CD279) on resting NK cells, IL-2 activated NKcells and PD-1 inhibited NK cells were detected by flow cytometry. Left figure: Expression of PD-1 on different processed NK cells. Red curve: NK cells with IL-2activated for 7 days; Blue curve: NK cells with PD-1 antibody inhibited for 24h. Green curve: resting NK cells; Brown curve: isotype. Right figure: Data were expressed as the mean ±SE.

The NK cells of C57BL/6 mice were characterized by the expression of the NK cell receptors NK1.1, CD49b, and NKp46 and the absence of CD3. As cultured NK cells are no longer recognized by DX5 antibodies in vitro [31], we chose the CD49b receptor to isolate the NK cells and used the other receptors as identifying markers. The NK cells isolated from mouse spleens were 90% CD3^-^ NK1.1^+^CD335^+^ based on flow cytometric analysis (Fig 2), which was consistent with a previous study [32].

### In vitro cytotoxicity of NK cells against GL261GSCs

**Fig 3:**
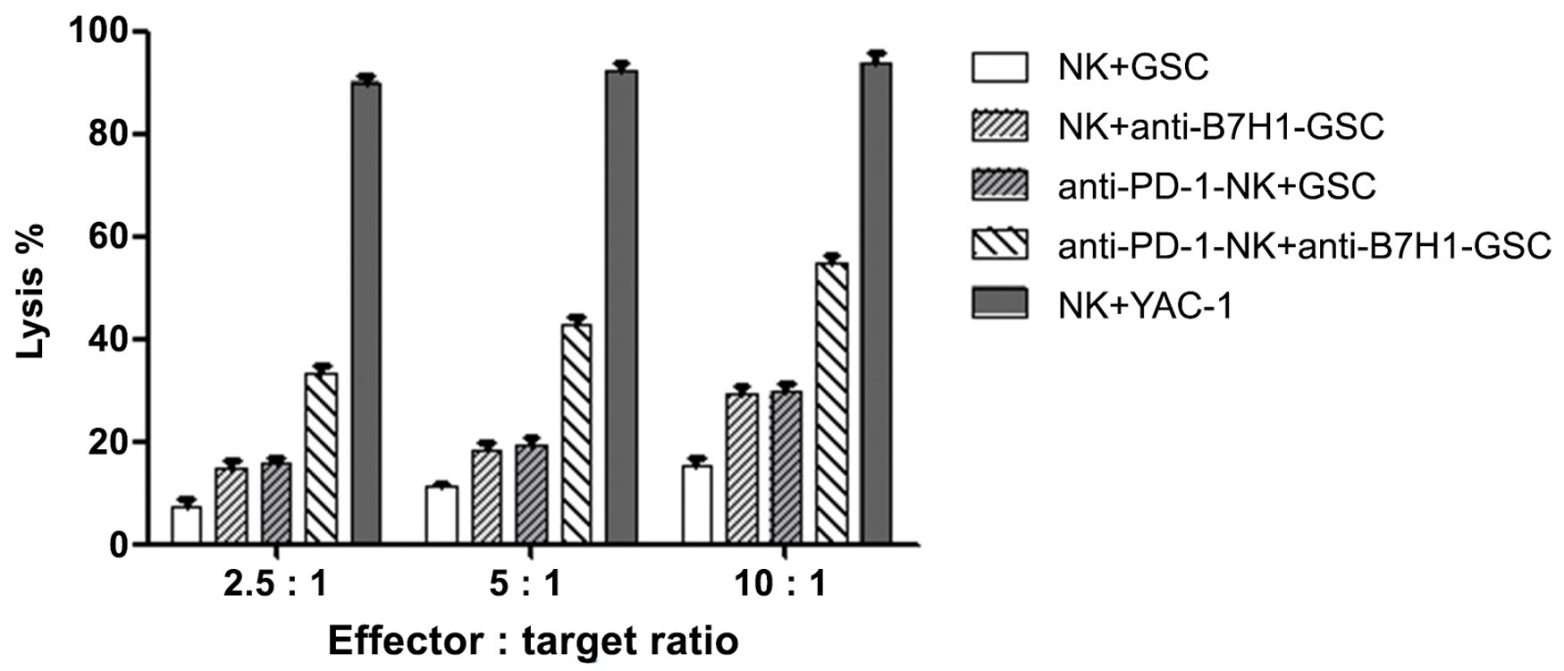
In vitro cytotoxicity of NK cells against GL261GSCs. Results of PD-1 blockaded and non-blockaded NK cells and B7H1 blockaded and non-blockaded GL261GSCs were combined into 4 groups. Cells were co-cultured for 6h. NK cells cytotoxicities against GL261GSCs in different groups were compaired by LDH assays. NK cells cytotoxity against YAC-1 were shown as a positive control. Data were expressed as the mean ±SE.

PD-1 inhibited and non-inhibited NK cells and B7H1 inhibited and non-inhibited GL261GSCs were combined into 4 groups at three specific E:T ratios (2.5:1, 5:1, and 10:1). After a 6-hour incubation, the NK-cell-mediated cytotoxicity against GL261GSCs was detected by an LDH assay. In the non-inhibited group, NK cells damaged 7.42%, 11.31%, and 15.1% of GL261GSCs at the following E:T ratios 2.5:1, 5:1, and 10:1, respectively. In the B7H1 inhibited group, the lysis percentages of GL261GSCs were 14.75%, 18.25% and 29.1%. In PD-1 inhibited group, the NK cells respectively destroyed 15.53%, 19.21% and 29.93% of GL261GSCs. Finally, in the double inhibited group, the lysis percentages were 33.24%, 42.86% and 54.91%. Despite whether the PD-1/B7H1 pathway was inhibited, the lysis percentage of GL261GSCs cells increased along with the increase of the E:T ratio. Furthermore, considering the same E:T ratio, the lysis percentage of the 4 groups were significantly different (E:T = 2.5:1, ANOVA F = 62.235, df = 3, P<0.001; E:T = 5:1, ANOVA F = 103.835, df = 3, P<0.001; E:T = 10:1, ANOVA F = 120.657, df = 3, P<0.001, Fig 3)). Next, we used the Bonferroni correction to compare the lysis percentages of the GL261GSCs between each group at the same E:T ratio. However, apart from the comparison between the B7H1 inhibited group and the PD-1 inhibited group, the lysis percentages of the 4 groups were significantly different from each other (Table 1).

**Table 1:** Comparison of lysis percentage between each group (Bonferroni correction).

E:T ratio	Group 1vs 2	Group 2 vs 3	Group 1vs3	Group 2vs4	Group 1vs 4	Group 3vs4
2.5:1	P = 0.033	P = 1.0	P = 0.017	P<0.001	P<0.001	P<0.001
5:1	P = 0.04	P = 1.0	P = 0.019	P<0.001	P<0.001	P<0.001
10:1	P = 0.001	P = 1.0	P = 0.001	P<0.001	P<0.001	P<0.001

Group 1: non-inhibited group. Group 2: B7H1 inhibited group. Group 3: PD-1 inhibited group. Group 4: double inhibited group.

### Therapeutic effect of NK cells in a GL261GSCs mouse model

**Fig 4:**
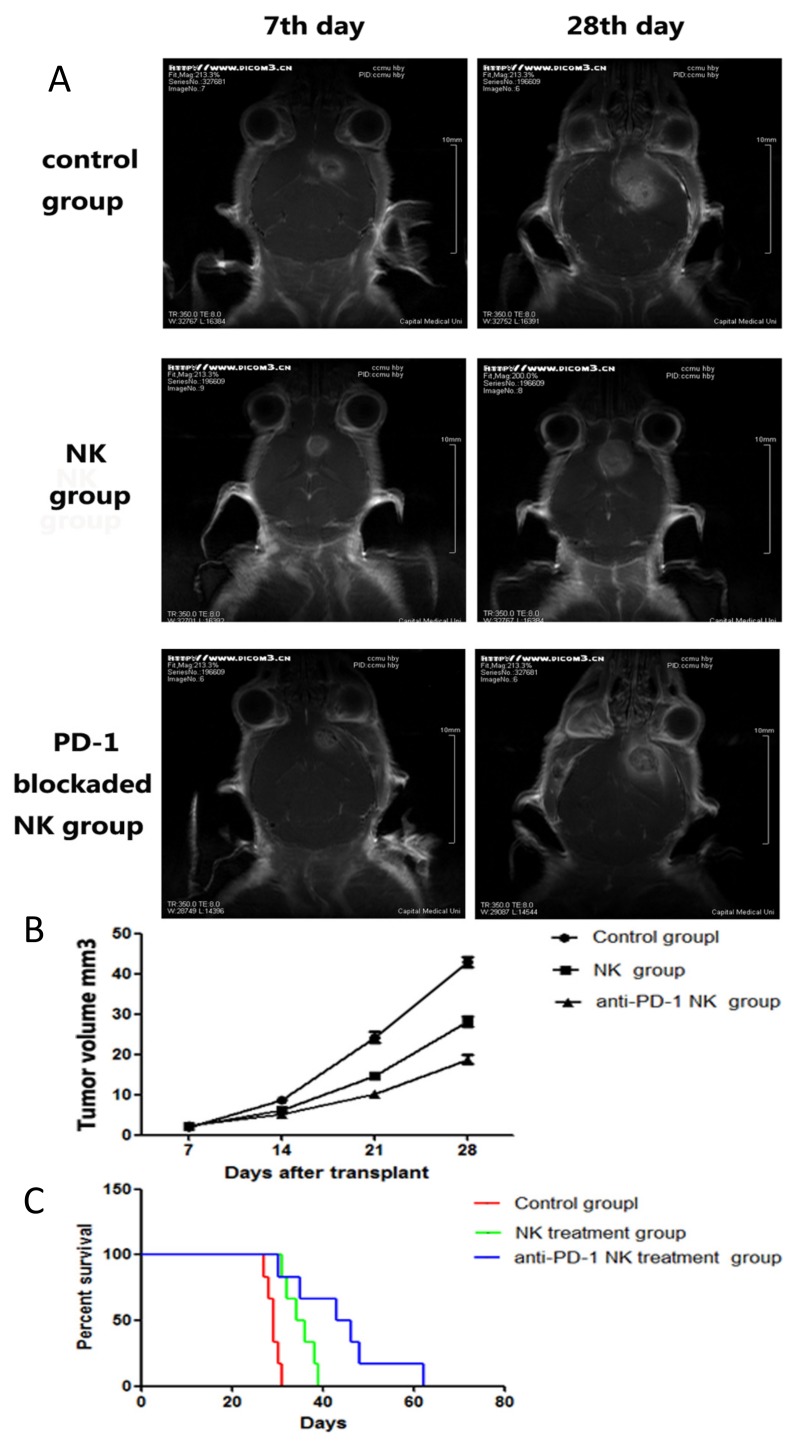
Therapeutic effect of NK cells against GL261GSCs mouse model. (A) Contrast-enhanced MRI of 3 distinct mice in the three treatment group before treatment (day 7) and after treatment were shown. (B) Tumor growth curves of three treatment groups were shown. Data were expressed as the mean ±SE. (C) Kaplan-Meier survival curves. Comparison of survival curves by log-rank (Mantel-Cox) test was statistically significant(P<0.001).

We used an anti-PD-1 antibody to verify the hypothesis that inhibition of the PD-1/B7H1 pathway can mediate a therapeutic effect in an orthotopic glioma stem-like cells mouse model. After implantation of 5×10^5^ GL261GSCs, tumor engraftment was confirmed with craniocerebral MRI scanning (on the 7th day after engraftment). Next, the mice bearing GL261GSCsGSCs were intravenously injected with PBS (negative control), 5×10^6^ IL-2-stimulated NK cells, or 5×10^6^ PD-1-inhibited IL-2-stimulated NK cells once a week for four weeks. MRI scanning was also performed after each injection. Representative images are shown for 3 distinct mice on the day 7 (before treatment) and day 28 (after treatment), fastest tumor growth was observed in the negative control group, while the mice in the PD-1-inhibited group had the slowest tumor growth. Treatment with non-PD-1-inhibited NK cells significantly inhibited GL261GSCs tumor growth compared with the control group (F = 118.9, P<0.01, Fig 4A). However, the tumor volumes showed a more obvious inhibition in the PD-1-inhibited NK cells treatment group (F = 308.5, P<0.01, Fig 4B). Additionally, the survival data substantiated the tumor growth patterns observed by MRI scans. The mice in the negative control group had a median survival of 29 days, and treatment with the non-PD-1-inhibited NK cells improved the median survival to 35 days (P = 0.0012, compared by log-rank survival curves). The median survival of the mice in the PD-1-inhibited group was prolonged to 44 days (P = 0.0024), with 1/6 of the mice achieving long-term survival (≥60 days after implantation, Fig 4C).

### In vivo treatment of NK cells decreased tumor cell proliferation and increased lymphocyte infiltration

**Fig 5:**
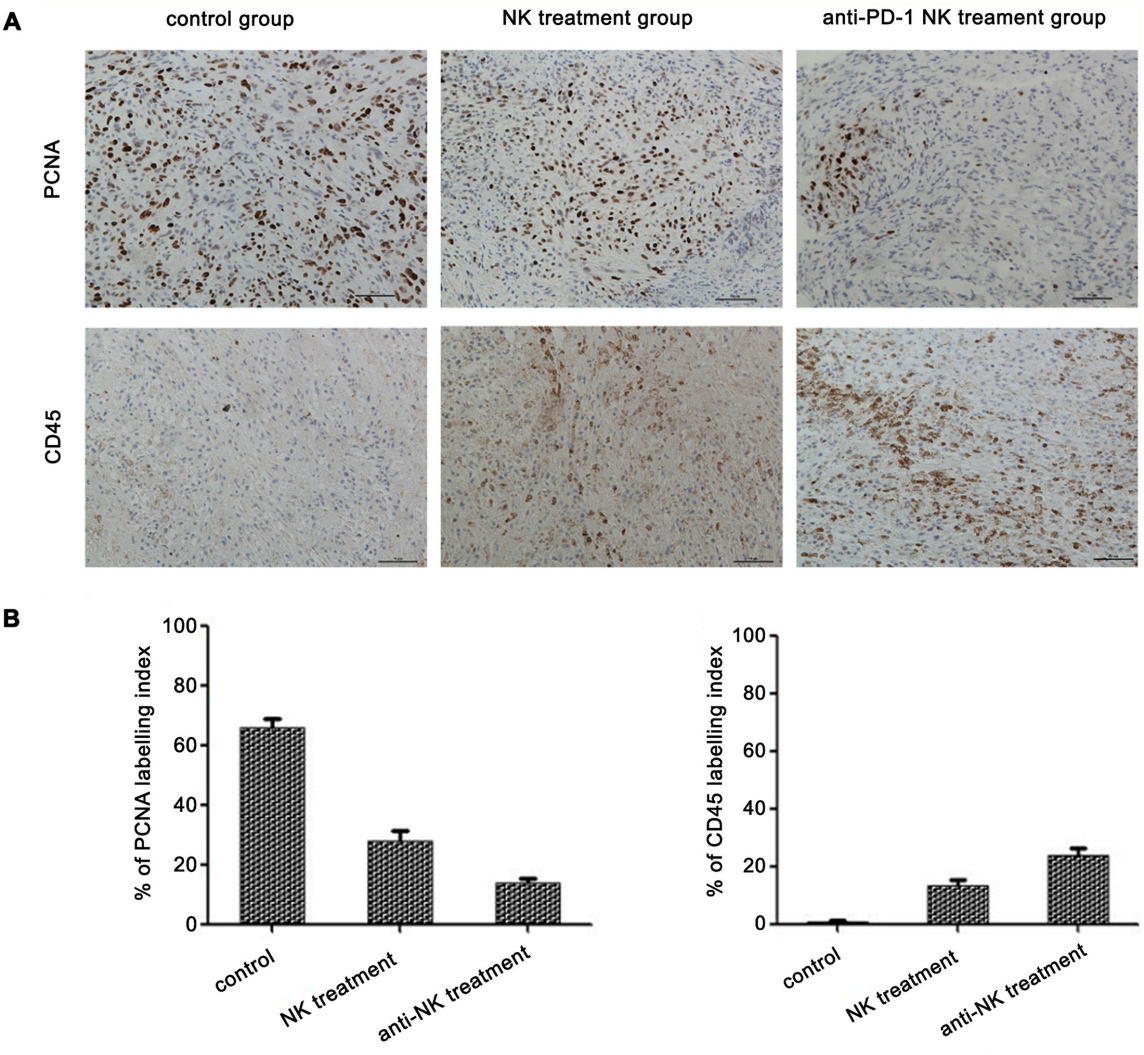
In vivo treatment of NK cells decreased tumor cell proliferation(PCNA) and increased lymphocytes infiltration(CD45). (A)Proliferating cells and tumor microenvironmet infiltrating lymphocytes were analyzed by anti-PCNA antibody and anti-CD45 antibody in tumor masses. (B) Percentage of PCNA positive and CD45 positive cells were compared (n = 5). Data were epressed as the mean ± SE.

We examined cell proliferation and lymphocyte infiltrating levels by immunohistochemical staining of PCNA and CD45, respectively (Fig 5). Compared with the PCNA-positive percentage of the control group (65.72%), the ratios in the PD-1 inhibited and non-inhibited NK cell treatment groups were 13.66% and 27.66% (F = 84.548, P<0.001), respectively. The two treatment groups were also significantly different (P = 0.016). Additionally, the number of CD45^+^ cells in the NK cell treatment group was 13.46% compared with 0.92% in the control group and 23.66% for the anti-PD-1 NK cell treatment group (F = 31.132, P<0.001). The two treatment groups were also significantly different (P = 0.012).

## Discussion

Traditionally, the PD-1/B7H1 pathway was thought to be a checkpoint to negatively regulate T-cell-mediated immune responses [33]. Tumor cells, which express PD-1 ligands, were thought to engage with PD-1 receptors, leading to T-cell exhaustion and tumor cells evaded immune response [21]. Accordingly, a series of clinical trials have been focused on using a PD-1 blocking antibody to enhance immunity in solid tumors [34–36] and hematologic cancers [37]. Alternatively, stimulated NK cells were reported to inhibit GBM in both clinical trials [24] and orthotopic GBM xenograft models [23,38]. Indeed, stimulated NK cells alone have not demonstrated efficacy as a primary therapy for GBM. In other words, glioma stem cells (GSCs) were a subset of glioma cells that possess the neural stem cells properties of self-renewal and multi-lineage differentiation which were supposed to play an essential role in in tumor initiation, metastasis, and recurrence. Accordingly, GSCs have become the most important target for GBM therapy. Tony Avril found that human GSCs could expressed lower level of the PD-L1 compared with the differentiated cells. And human GSCs were more sensitive to the cytotoxity of the IL-activated NK cells [39]. Our flow cytometry results of the B7H1 expression on the GL261GSCs and differentiated GL261 cells were in accord with Tony's research. Accordingly, we belived that the lower level of the B7H1 expression may contribute to the more sensitivity of GSCs to cytotoxity of the IL-activated NK cells, and thus make GSCs as a more suitble target for immunotherapy. Combining the negative immune regulation of the PD-1/B7H1 pathway [33] and the anti-GSCs effect of stimulated NK cells, we hypothesized that blockade of the PD-1/B7H1 pathway between NK and GSCs might interrupt immunosuppression and promote NK cells killing GSCs. Our hypothesis was substantiated by the cytotoxicity assay. Notably, simultaneous inhibition of PD-1 and B7H1 achieved the most efficient co-toxicity of NK cells against GL261GSCs. Although a single blockade of the receptor (PD-1) or the ligand (B7H1) demonstrated no significant difference in the cytotoxicity assay, the effect was still more potent than that observed in the non-inhibited group.

Previous studies have shown that the inhibition of PD-1 on CTLA-4 promote tumor regression and prolong survival in both extracranial cancer models [40, 41] and clinical trials [37, 42]. For the first time, we demonstrated that inhibition of the PD-1/B7H1 pathway promotes the co-toxicity of NK cells against GSCs in vitro. In the intracranial GSCs model, mice that received PD-1-inhibited NK cell treatment showed both longer survival and slower tumor growth. Furthermore, the mouse that achieved long-term survival showed neither obvious body weight loss nor distinct neurological deficiencies in movement or feeding. Considering the mortality and morbidity of GBMs, these results demonstrate that PD-1-inhibited NK cells may be an effective and promising approach for GBM immunotherapy. Moreover, in our vivo experiments, we confirmed that not only could the anti-PD-1 NK cell treatment inhibit the proliferation of GBMs, but more importantly, the intravenous injection of PD-1-inhibited NK cells could promote the infiltration of lymphocytes into the intracranial tumor microenvironment. Notablely, these infiltrating lymphocytes may be derived from the PD-1-inhibited NK cells injected into the mice blood circulation. Another possible source is the immune regulation of the PD-1-inhibited NK cells that promoted other lymphocytes to accumulate in the brain. Thus, further studies need to be done to investigate the specific constitute and source of the infiltrating lymphocytes.

Traditionally, studies have focused on regulating the function of CTLs to act as an immunotherapy against GBM. However, although CTLs have specific anti-tumor effects, the tumor cells can still evade recognition by down-regulating the expression of MHC-I. Adoptive immunotherapy is dedicated to taking advantage of cytokine-induced killer cells (CIK cells) and lymphokine-activated killer cells (LAK cells) to treat GBM. For CIK cells, human PBMCs are traditionally required to be incubated for 4 weeks with numerous cytokines to generate CIK cells [43]. Moreover, LAK cells, as immunotherapeutic agents, also have some disadvantages, such as a short in vivo lifespan, poor cytotoxicity, and numerous side effects [44–45]. Accordingly, the PD-1-inhibited NK cells, with direct activation and potent cytotoxicity, lack of MHC-restriction, and most importantly, no PD-1-mediated immunosuppression, could be a more feasible immune therapeutic approach against GBM.
